# Cellular lineage origins of spasmolytic polypeptide-expressing metaplasia (SPEM): persistent and intensifying debates

**DOI:** 10.3389/fonc.2025.1642559

**Published:** 2025-10-21

**Authors:** Xiaofeng Li, Yu Li, Lili Wu, Jingbin Wang, Guoxin Huang, Lei Rong, Wenjuan Shen, Liang Ma, Yang Zhang

**Affiliations:** ^1^ First Clinical Medical College, Heilongjiang University of Chinese Medicine, Harbin, China; ^2^ Department of Oncology, First Affiliated Hospital of Heilongjiang University of Chinese Medicine, Harbin, China; ^3^ Department of Spleen and Stomach Diseases, Shenzhen Hospital (Fu Tian) of Guangzhou University of Chinese Medicine, Shenzhen, China; ^4^ Department of Gastroenterology, First Affiliated Hospital, Heilongjiang University of Chinese Medicine, Harbin, China; ^5^ Department of Obstetrics and Gynaecology, First Affiliated Hospital, Heilongjiang University of Chinese Medicine, Harbin, China

**Keywords:** spasmolytic polypeptide-expressing metaplasia (SPEM), gastric cancer, metaplasia, cellular lineage, precancerous lesion

## Abstract

Spasmolytic Polypeptide-Expressing Metaplasia (SPEM) is a gastric fundic gland metaplasia resembling deep antral glands, associated with drug injury, *Helicobacter pylori* (*H. pylori*), or bile reflux. Early-stage SPEM acts as a reparative response, but if the damaging stimuli persist, the metaplastic changes may become irreversible, raising the risk of gastric cancer development. Traditionally, SPEM arises via passive transdifferentiation of chief cells following parietal cell loss. However, recent lineage tracing and genetic models challenge this, suggesting active depletion of chief cells and involvement of isthmus stem cells also contribute to SPEM development, intensifying debate over its cellular origins. This review synthesizes SPEM’s physicochemical drivers and critically evaluates evidence for the three proposed sources: (1) passive chief cell transdifferentiation (2), active chief cell loss, and (3) isthmus stem cells. Clarifying the heterogeneity in the origin of SPEM is challenging until more specific cell ablation techniques are developed, but timely classification of existing research may be instructive.

Gastric cancer is a prevalent global health issue, and its prognosis is closely associated with early diagnosis. Actively intervening in or blocking the pathological progression of precancerous gastric lesions represents an effective strategy for gastric cancer prevention and treatment. The malignant transformation of gastric mucosa is closely associated with precancerous events such as gastric mucosal atrophy (parietal cell loss) and glandular metaplasia (1). Numerous studies suggest that metaplastic lesions—including intestinal metaplasia (IM) and spasmolytic polypeptide-expressing metaplasia (SPEM) (2)—may represent the final reversible stage in the Correa cascade leading to intestinal-type gastric cancer (3–5). SPEM is a reparative cellular lineage in the gastric corpus glands, characterised by the metaplastic transformation of chief cells, mucous neck cells, or isthmus stem cells into spasmolytic polypeptide-secreting mucinous cells (6–9). This essentially represents a product of dysregulated gastric mucosal renewal, cellular reprogramming or transdifferentiation induced by inflammatory microenvironmental factors (10–12). SPEM is defined by co-expression of multiple markers, including GS-II lectin, CD44 variant 9 (Cd44v9), human epididymis protein 4 (HE4), aquaporin 5 (AQP5), gastrokine 3 (Gkn3), trefoil factor 2 (TFF2), and mucin 6 (MUC6) (13–19). As the initial stage and critical pathological component of gastric epithelial metaplasia, SPEM has emerged as a new research focus in precancerous lesion studies, though its precise pathogenic mechanisms remain elusive (20). Recent advances in research methodologies have yielded progressive insights into the cellular origins of SPEM. Based on the architectural features of gastric mucosal glands, experimental *in vivo* and *in vitro* models, and the role of SPEM in gastric epithelial malignant transformation, this review synthesizes research on the cellular lineage origins of SPEM, offering insights into the biological characteristics of this metaplastic lesion.

## 1 The relationship between SPEM, intestinal metaplasia, and gastric cancer

SPEM is currently a key area of investigation in molecular and cellular biology, being considered as a potential cellular origin for IM, dysplasia, and gastric adenocarcinoma (21–23). SPEM was first discovered by Wang et al. in the gastric fundic mucosa of mice infected with feline-derived *H. pylori*. It arises predominantly at the base of the glands in the gastric fundus or corpus, demonstrating phenotypic features resembling antral gland and Brunner’s gland differentiation. SPEM cells exhibit a unique molecular signature characterized by specific expression of Trefoil Factor 2 (TFF2) and Mucin 6 (MUC6) (24–26). In contrast, the more extensively studied IM presents classical intestinal differentiation markers, including goblet cells and Paneth cells, along with characteristic expression of Trefoil Factor 3 (TFF3) and Mucin 2 (MUC2) (27, 28). SPEM is considered to be the precursor stage to IM (29–31). In double-staining observations of human gastrectomy specimens, SPEM cells were observed in the basal region of the gastric mucosa and were detected to be positive for both PAS and TFF2 staining. In the luminal part above the SPEM, goblet cells positive for Alcian Blue and Muc2 staining were observed. Ki67+ cells were sparsely located in the SPEM area and were mostly in the Muc2- positive area adjacent to the SPEM. These results reflect that the enhanced proliferative activity of IM may be the result of further differentiation of SPEM and supports the hypothesis that SPEM is a key initial premalignant metaplasia associated with gastric adenocarcinoma (32). For over a century, researchers have repeatedly documented the progressive proximal migration of atrophic and metaplastic changes from the antral glands toward the gastric corpus. This phenomenon formed the pathological basis for the Kimura-Takemoto endoscopic classification system (31, 33). However, scientific interest has disproportionately focused on IM rather than SPEM, likely due to endoscopic sampling limitations. Routine biopsy protocols typically obtain only superficial tissue through random, targeted sampling, consequently missing the deeper glandular compartments where SPEM predominantly initiates (34).

The malignant transformation potential of SPEM primarily stems from its pronounced genetic instability (35). The metaplastic conversion of pepsinogen-producing cells to mucous cells emerging shortly after mucosal injury is generally regarded as a multi-step process of epithelial restitution. This dynamic remodeling mechanism constitutes an adaptive repair response in gastric mucosa, where specialized secretory cells undergo phenotypic switching to restore barrier integrity (32). Compared to normal tissue, metaplastic mucosa seems to have a stronger ability to resist potential inflammatory injury (10). However, persistent damage and chronic inflammation can lead to the permanent establishment of recurrent reprogramming and metaplasia patterns, which poses a risk for gastric cancer development (36, 37). SPEM lineage can be found in over 80% of resected samples of gastric adenocarcinoma and residual gastric mucosal tissue, and SPEM glands can also be found in most dysplasia tissues (20, 21). A comprehensive genomic landscape evaluation employing exon sequencing on commonly dysregulated genes in intestinal-type gastric carcinoma revealed striking molecular convergence. Pathogenic missense mutations in MUC5AC, KRAS, BRAF and EZH2 exhibited significant overlap between SPEM and intestinal-type gastric cancer. Notably, the mutant alleles showed a clear trend of gradual accumulation during disease progression to dysplasia and gastric cancer. This mutational continuum provides compelling evidence for the clonal evolutionary trajectory linking SPEM to the development of dysplasia and gastric adenocarcinoma (31, 38). Even some studies suggest that SPEM exhibits a more robust pathobiological correlation with gastric adenocarcinoma in gastric carcinogenesis when contrasted with IM (20, 23). It is worth noting that although the correlation between SPEM and intestinal tumors has been partially validated, the precise cellular identity driving the development of SPEM remains a mystery, and its graded differentiation trajectory within the gastric gland remains uncertain.

## 2 Factors contributing to SPEM

### 2.1 The interplay between *H. pylori* infection and SPEM

Chronic *H. pylori* infection not only results in severe acid-gland atrophy and drives SPEM development (39, 40), but also leverages inflammation-related metaplastic changes to expand its niche through spatial colonization. In this process, the initial event is the colonization of *H. pylori*, altering the distribution of gastric microbiota and the abundance of proteins. By influencing microbial metabolism, *H. pylori* secretes effector proteins and toxins, including CagA and VacA (41, 42), disrupting gastric cell junctions and the polarization of apical-basal cells. Further activation of pro-inflammatory and carcinogenic signaling pathways leads to impaired integrity of gastric epithelium, disrupted cell differentiation and disturbed self-renewal, ultimately resulting in inflammatory response (43). Chronic damage to the gastric mucosa can induce the recruitment, homing, and proliferation of bone marrow mesenchymal stem cells (BMSCs) under inflammatory stimulation, further becoming a prerequisite for dysplasia and cancer (44, 45). Inflammation-mediated immune cell infiltration is an important mechanism and broad background for the occurrence of SPEM (46).

SPEM-induced mucosal remodeling creates more suitable conditions for *H. pylori* colonization. *H. pylori* mainly colonizes by binding its two adhesins Bab and Sab to glycosylation receptors Lewis B (Leb) and sialylated Lewis X (sLex) in host epithelial cells. Compared to intestinal metaplasia, SPEM can specifically enhance sLex expression, which to some extent determines the colonization and diffusion of *H. pylori* towards the proximal part of the gastric body and deeper glandular layers. Following the onset of SPEM, *H. pylori* can indirectly promote the spread of SPEM throughout the stomach by inducing this metaplastic repair response via chronic inflammation, or directly promote the spread of SPEM throughout the stomach by secreting toxins such as CagA that affect epithelial differentiation (47). In low gastric acid conditions, *H. pylori*’s tendency to target SPEM increases, further enhancing its colonization, adhesion, and invasion abilities, resulting in persistent inflammation and the further establishment of a tumor microenvironment.

SPEM caused by *H. pylori* infection presents as chronic lesions, most of which are irreversible. *H. pylori* utilizes its motility, chemotaxis, toxin production, and other mechanisms to adapt to the acidic conditions of the gastric lumen (48), thereby evading immune recognition and causing persistent inflammation. Continuous damage and repair induce abnormal cell proliferation, directly increasing DNA replication pressure at the genetic level (49), promoting double strand breaks (DSBs) (50), and overloading the DNA damage response (DDR) pathway (51). This response is normally triggered by signalling damage, repairs cells in an error-free manner and causes apoptosis and senescence. This prevents malignant progression in the presence of incorrectly repaired DNA, which can lead to tumour formation when the DDR signalling pathway is inefficient due to overload and DNA repair is compromised. Eradication of *H. pylori* reverses DDR activation, but not cellular senescence. An increased number of senescent cells in the area of metaplasia acts as a carcinogen and promotes disease progression further (52). This could explain why *H. pylori* eradication is ineffective in preventing gastric cancer in the presence of precancerous lesions such as IM (53, 54).

### 2.2 Drug impairment due to tamoxifen, DMP-777, L-635

Current animal models of SPEM are mostly induced and established using high doses of drugs such as tamoxifen, DMP-777, and L-635 (55, 56), which directly cause an acute decrease in the number of gastric lining cells and the development of SPEM within 3–14 days through drug toxicity effects. Among them, tamoxifen belongs to the selective estrogen receptor modulators, which are mostly used in breast cancer chemotherapy or as inducers involved in gene editing. A single dose of tamoxifen ≥ 3 mg/20 g body weight given to normal mice for 3 days resulted in apoptosis of more than 90% of the parietal cells and metaplasia of the chief cells (57). Comparative studies utilising wild-type and gastrin-deficient animal models have demonstrated that SPEM-induced oxyntic gland atrophy occurs through gastrin-independent mechanisms (56). Notably, findings from Manning et al. (58) confirmed this observation, revealing that tamoxifen acts as a protonophore by disrupting the proton gradient across the acid-secretory membrane of parietal cells. This disruption to acid-base homeostasis induces parietal cell apoptosis, a process thought to involve muscarinic receptor-dependent pathways. DMP-777 also exhibits protonophore activity but does not affect the activity of H^+^/K^+^-ATPase (59). Additionally, as a neutrophil elastase inhibitor, DMP-777 induces the development of SPEM by reducing the degradation of extracellular matrix components such as elastin and collagen. This preservation of matrix integrity thereby prevents the onset of inflammation. L-635 is a prototype carrier analog of DMP-777. Due to its lack of elastase-inhibitory activity, treatment of mice with L-635 can establish a parietal cell depletion model accompanied by acute inflammatory responses. This intervention further promotes the transition of the SPEM lineage from relatively inert metaplasia to a proliferative metaplastic phenotype, ultimately leading to the development of dysplasia (6). The feature is similar to the chronic SPEM model established in cats infected with *H. pylori*.

### 2.3 Bile reflux and immunological factors

**Figure 1:**
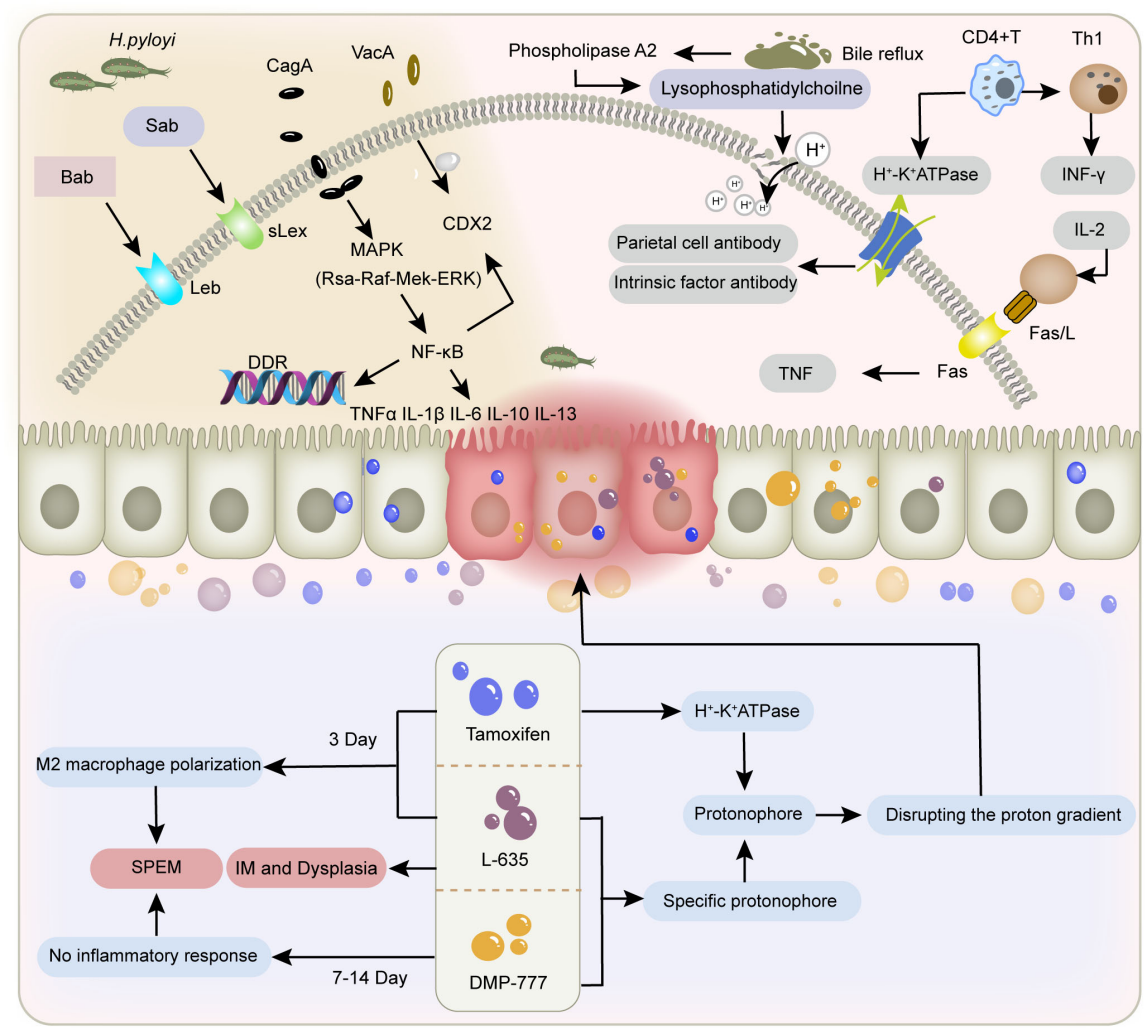
Factors contributing to gastric mucosal injury. The figure depicts the different mechanisms of gastric mucosal damage caused by *H. pylori* infection, bile reflux, immune factors, and tamoxifen, L-635, and DMP-777.

In addition to *H. pylori* infection and drug-induced damage, bile reflux and immune dysregulation also contribute to the development of SPEM (60). During bile reflux, bile phosphatidylcholine is hydrolyzed into lysophosphatidylcholine by phospholipase A2, resulting in degradation of the phospholipid layer in gastric mucosal epithelial cells. This disruption of the gastric mucosal barrier facilitates the back-diffusion of H^+^ into the submucosa (61), thereby altering the microenvironment critical for parietal cell survival. Furthermore, bile acids inhibit nitric oxide synthase (NOS) activity and impair cellular H^+^/Na^+^ exchange (62), while modifying DNA methylation patterns (63, 64). These cumulative effects induce DNA damage, apoptosis, and mutagenesis in gastric mucosal cells, ultimately driving mucosal atrophy and metaplasia. Immune-mediated SPEM predominantly emerges during the pathological progression of autoimmune gastritis. Autoimmune gastritis is an autoimmune disorder characterized by autoimmune attacks against parietal cells and intrinsic factor, with SPEM representing one of the most frequent metaplasia subtypes observed in this condition (65). CD4^+^ T cells trigger an autoimmune response targeting the H^+^/K^+^-ATPase on the secretory canalicular membranes of parietal cells. This immune-mediated destruction leads to parietal cell loss and atrophy of the gastric glands, representing a core mechanism of autoimmune-related parietal cell depletion. A more specific mechanism may involve Fas ligand-induced activation of the extrinsic apoptosis pathway (66, 67) (**Figure 1**).

## 3 Controversies surrounding the origin of SPEM

### 3.1 Prevailing hypothesis: parietal cell loss triggers transdifferentiation of chief cells

Chief cells are functional cells located at the base of gastric fundic glands, responsible for secreting pepsinogen granules. They express stem cell molecular markers muscle intestine and stomach expression 1 (Mist1), tumor necrosis factor receptor superfamily member 19 (Troy), and leucine-rich repeat-containing G-protein coupled receptor 5 (Lgr5), and can act as reserve stem cells *in vivo (* 68, 69). These cells normally maintain homeostasis of themselves and other gastric gland cells through slow turnover (70). Once injured, chief cells change their transcriptional signature to increase repair of damaged tissue (71). A reduction in parietal and chief cell numbers is observed in most SPEM animal models, with the highly plastic chief cells being recognized as the cellular origin of metaplasia (6). Caldwell et al. (72) developed novel chief cell-specific GIF-trTA allele mouse models for lineage tracing. In this study, GFP tracing markers were initially detected exclusively in the basal gland regions housing chief cells, showing co-expression only with chief cell markers (GIF) and SPEM markers (CD44v9, TFF2, GSII). This co-expression pattern significantly diminished after 12 months, while GIF-negative GFP-labeled cells became scattered in gastric corpus glands, correlating with Ki67 (proliferative cells), UEAI (surface cells), GSII (mucous neck cells), and H/K-ATPase (parietal cells) positive populations. These findings indicate that during the longer survival cycle, subsets of mature chief cells exhibit reserve stem cell properties to transdifferentiate into various lineages, resulting in self-depletion, thus providing direct evidence for chief cells’ regenerative potential in gastric glands. Conventional lineage tracing approaches are fundamentally limited by the temporal imprecision of Cre-loxP systems, including both promoter activation delays and reporter expression lag. These technical constraints restrict detection to initial progenitor populations and terminal differentiated states, precluding discrimination between transdifferentiation and dedifferentiation pathways. In contrast, single-cell sequencing technologies enable high-resolution trajectory reconstruction by computationally ordering cells based on transcriptional profiles, thereby revealing both linear differentiation cascades and branched fate decisions.A Monocle pseudotime trajectory analysis of representative chief cell genes (73) has for the first time delineated their potential differentiation pathways at single-cell resolution, demonstrating that chief cells can transdifferentiate into mucous neck cells before progressing to SPEM, strongly supporting the chief cell origin hypothesis of SPEM.

The loss of parietal cells has been identified by multiple research groups as a critical precursor to chief cell transdifferentiation following gastric mucosal injury (14, 74). In gastric parietal cell-specific solute carrier family 26 member 9 (Slc26a9) knockout mouse models (Slc26a9fl/fl/Atp4b-Cre) (75), pyroptosis-driven parietal cell loss and SPEM development can be observed. Similar findings have been replicated in other animal models. However, it remains undetermined whether SPEM arises through proliferative differentiation or direct transdifferentiation of chief cells post-parietal cell depletion. Subsequently, the Nam research team (6) developed Mist1CreER/+/Rosa26RLacZ mice using the CRISPR/Cas9 gene-editing system for novel lineage tracing studies. In this model, tamoxifen-induced Cre was knocked into the chief cell-specific Mist1 locus, leading to β-galactosidase expression upon chief cell maturation. Observation of this reporter gene revealed that SPEM cells exhibiting β-galactosidase activity emerged in the gastric fundic mucosa across three parietal cell-depletion models: DMP-777 (non-inflammatory), L-635 (acute inflammation), and *H. pylori* infection (chronic inflammation). Notably, enhanced β-galactosidase activity and accelerated SPEM progression were observed in inflammatory models. These findings suggest that under conditions of significant parietal cell reduction, SPEM originates predominantly from mature chief cell transdifferentiation, and that the combination of parietal cell loss and inflammation synergistically promotes SPEM development. In another model using 5-fluorouracil to block mitotic division in gastric mucosal cells (8), tamoxifen was still able to induce parietal cell loss and SPEM formation in mice with suppressed proliferation. It was inferred that some SPEM formation may be related to direct reprogramming of existing basal chief cells, not dependent on mitotic genetic mechanisms.

The aforementioned research demonstrates from two aspects that SPEM occurrence is temporally and spatially related to chief cell transdifferentiation induced by parietal cell loss (1): Gastric epithelial metaplasia consistently emerges following parietal cell atrophy. Autoimmune gastritis (76), chronic *H. pylori* infection (22), acute drug induction (55, 56), and novel parietal cell-specific knockout models (72, 75) collectively confirm that mature parietal cells are crucial regulators of gastric epithelial differentiation. After oxyntic gland atrophy, the phenotypic characteristics of emerging SPEM cells resembling chief cells suggest their origin from parietal cell loss-induced chief cell alterations (77) (2). Subsequent lineage tracing (6) and immunohistochemical evidence (8) have robustly demonstrated chief cells’ multidirectional differentiation potential through longitudinal observation and multi-model validation. Critically, these investigations established that chief cells can directly transdifferentiate into SPEM cells independent of proliferative activity, challenging the conventional paradigm of mitosis-dependent metaplasia. Similar mechanisms are observed in tumor epithelial-mesenchymal transition (EMT), where gastric epithelial cells lose polarity and acquire stem-like properties through coordinated epigenomic and genomic changes, entering mesenchymal states with enhanced migratory/invasive capacities. Although enhanced proliferative activity may coexist with EMT in tumor cells, these processes function synergistically (78, 79). This provides insights for understanding SPEM-associated chief cell transdifferentiation, revealing that SPEM essentially represents cellular plasticity akin to EMT (80). The collective significance of the aforementioned studies lies in establishing the central role of chief cell plasticity in the context of parietal cell loss in SPEM formation. However, they have not yet precisely unraveled the dynamic process of cell fate transition. To uncover more intuitive and profound regulatory mechanisms, it is essential to establish a more accurate functional research system for SPEM, which requires further development of animal models with permanently induced parietal cell atrophy, the advancement of lineage tracing technologies with higher spatiotemporal resolution, and the application of multi-omics analyses.

### 3.2 New perspective: active loss of chief cells independent of parietal cell ablation can also induce SPEM

Previous studies predominantly focused on parietal cell loss as the initiating factor driving chief cell transdifferentiation. However, research involving targeted ablation of parietal cells failed to induce SPEM, challenging the prevailing view. This evidence indicates that parietal cell loss serves as a permissive condition rather than a driver for SPEM development (81). Emerging evidence from chief cell-specific gene knockout models and pharmacogenetic approaches supports the perspective that active depletion of chief cells, even in the presence of intact parietal cells, can lead to SPEM and subsequent gastric epithelial malignant transformation (69, 82, 83).

Runt-related transcription factor 3 (RUNX3), a pivotal developmental regulator, integrates microenvironmental signals to modulate cell cycle progression and cell fate determination (84). In the BALB/c background RUNX3^-/-^ adult mouse model established by Ito et al. (83), researchers observed loss of chief cells and SPEM development without inflammatory intervention, while parietal cells remained intact. This model subsequently developed gastric adenocarcinoma, indicating that mere parietal cell atrophy is insufficient to fully induce metaplasia and that SPEM may arise through mechanisms beyond parietal cell injury or death. However, prior studies have established that RUNX3 dysregulation disrupts cellular differentiation and proliferative homeostasis, driving intestinal-type gastric carcinogenesis (85, 86). Thus, the above findings only confirm that RUNX3 deficiency induces SPEM independently of parietal cells, with chief cell loss being a critical event in this process. Since the RUNX3^-/-^ model targets the entire gastric epithelium (not chief cell-specific), it cannot directly support the notion that primary chief cell depletion initiates SPEM. Subsequently, Liu et al. (82) demonstrated that CHIA is highly expressed in chief cells, and its loss in chief cells constitutes a pivotal event driving SPEM development and gastric cancer progression independently of parietal cell loss. To validate whether primary chief cell depletion directly induces SPEM, the team established a chief cell-selective CHIA-deficient mouse model (CHIA^rox/rox^-GIF-Dre) using Dre-Rox technology. In this model with intact parietal cells but depleted chief cells, they observed upregulated expression of SPEM markers (Wfdc2 and CD44v9) and loss of the chief cell marker MIST1, accompanied by reduced expression of the stemness marker Lgr5. The parietal cell marker ATP4b showed only minor changes. Electron microscopy further revealed relocalization of the mucous neck cell marker SRY-box transcription factor 9 (SOX9) from the isthmus-neck region to the gastric corpus, with co-localization of SPEM markers TFF2 and CD44v9. These findings suggest that in the presence of parietal cells, chief cells may transdifferentiate into mucous neck cells, thereby inducing SPEM. However, the Barker group (69) proposed a different view on which kind of cells the exhausted chief cells transdifferentiate into. The team selectively ablated Lgr5^+^ chief cells (accounting for 40% of all chief cells) using diphtheria toxin in the Lgr5-DTR-EGFP mouse model, revealing that damage to this subset drives gastric epithelial regeneration. Furthermore, upon tamoxifen-induced Kras (G12D) mutation (Lgr5-2A-CreER-T2^tg/tg/^LSL-Kras(G12D)^tg/+^), these cells directly progressed to gastric cancer. This evidence indicates that Lgr5^+^ chief cells are prone to malignant transformation and can serve as the cellular origin of gastric cancer. Thus, the study proposes that under physiological conditions, Lgr5^+^ chief cells function as terminally differentiated cells; however, upon gastric tissue injury, they acquire stem-like properties, participating in SPEM formation and initiating malignant transformation.

Previous studies have revealed that gastric chief cells are dynamically regulated by p57 and can coordinate tissue homeostasis and damage repair in response to microenvironmental changes (87). Current evidence (69, 82, 83) further reveals a dual-pathway role for chief cells in SPEM pathogenesis: SPEM may arise partially through secondary responses to parietal cell loss; partially through gene mutations induced by active chief cell depletion. Subsets of chief cells marked by distinct molecular signatures may exhibit fate heterogeneity during transdifferentiation: some differentiate into mucous neck cells, while others acquire stem-like properties—yet both pathways drive SPEM development. Nevertheless, additional evidence is required to validate the chief cell hypothesis. Current research disproportionately focuses on the entire gastric corpus gland rather than specifically on its basal region (the chief cell zone). Future studies involving targeted ablation of mucous neck cells and isthmus stem cell depletion hold crucial implications for delineating the temporal dynamics of SPEM progression and elucidating its underlying mechanisms.

### 3.3 Additional controversies: SPEM arises from gastric stem cells

It is generally accepted that multipotent stem cells in the gastric isthmus are rapid-cycling stem cells that govern cellular differentiation in gastric glands and mediate tissue repair (88, 89). Stem cells generate lineage-specific progenitor cells that differentiate into pit cells at the gland apex, maintain stem cells and parietal cells in the isthmus, and form neck mucous cells in the neck region. These neck mucous cells subsequently migrate along the basal region and progressively differentiate into chief cells (90, 91).Over the past three decades, the consensus kinetic model of gastric epithelial and chief cell dynamics proposes that upon activation, isthmus-derived stem cells traverse specific migratory pathways along the neck region. Through intermediate transitional stages involving pre-neck mucous cells and pre-chief cells, they ultimately undergo transdifferentiation into mature chief cells (92–94). This conceptual framework has established the theoretical basis for the stem cell/pre-metaplastic phenotype origin hypothesis of SPEM.

Previous studies on SPEM predominantly relied on lineage tracing, identifying cellular changes through expression domains of molecular markers, which partially elucidated spatial distribution patterns of local cell populations. Although the expansion of TFF2/GIF double-positive cells and lineage tracing using GIF/Mist1 support chief cell origins, some scholars contend that the transdifferentiation hypothesis remains flawed (1): Earlier studies assumed Mist1, Troy, and Lgr5 as chief cell-specific markers, yet Mist1 and Troy also label isthmus stem cells (95, 96), while Troy is expressed in parietal cells (68). Although the Lgr5-2A-CreERT construct demonstrates relative chief cell specificity (69), evidence shows Lgr5 mRNA expression in the isthmus post-injury, inducing genetic recombination in Lgr5-EGFP-IRES-CreERT mice (9) (2); Lineage tracing models primarily employ tamoxifen-induced Cre activation, yet TAM concurrently induces parietal cell death, epithelial cell apoptosis, and impairs stem cell activity (57, 97); (3) Troy-CreERT knock-in mouse studies proposed chief cells possess “reserve” stem cell properties (68), but observed behaviors were later confounded by Troy haploinsufficiency (95); (4) Most models utilize drug-induced acute SPEM, which differs from chronic genetically-reprogrammed SPEM in morphology and molecular mechanisms, failing to represent authentic metaplasia (98).

**Figure 2:**
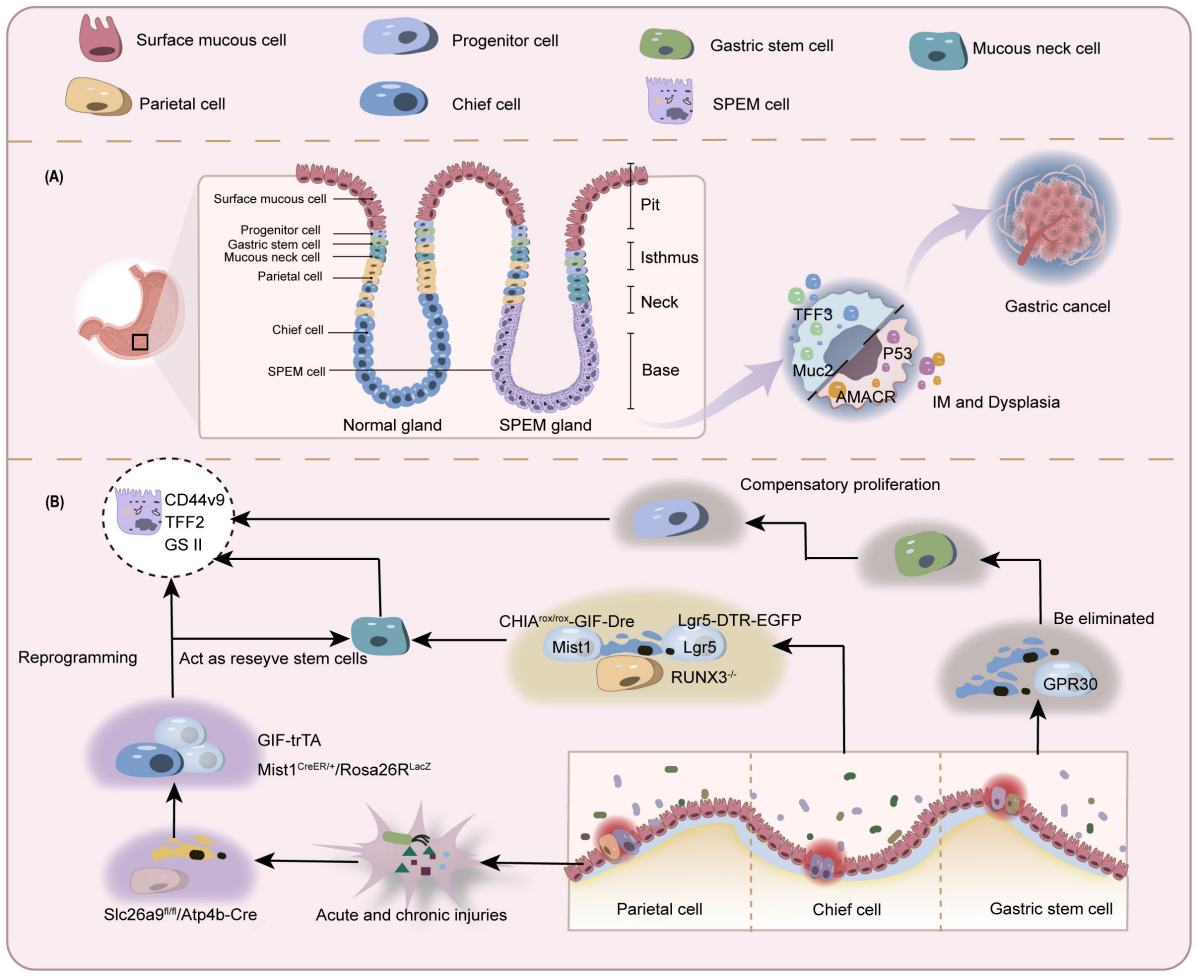
Schematic of controversial origins of SPEM. **(A)** Normal gastric glands comprise various cell types including but not limited to chief cells, parietal cells, stem cells, and mucous neck cells. In the context of gastric mucosal injury with depletion of parietal and chief cells, SPEM cells emerge in the basal gland region and may progress toward IM and gastric cancer. **(B)** In parietal cell loss-induced chief cell transdifferentiation: A subset of chief cells undergoes direct reprogramming into SPEM cells. Another subset differentiates into mucous neck cells before developing into SPEM. Similar bifurcation occurs during active chief cell transformation. The stem cell origin hypothesis proposes depletion of chief cells prior to SPEM development, with true origins residing in progenitor/stem cells.

Hayakawa, a leading proponent of skepticism, addressed these limitations by developing an alternative lineage tracing system (99). Bypassing conventional Cre-based constructs, his team crossed TetO-Cre mice with Gpr30-rtTA mice to specifically label GPR30+ chief cells, coupled with fluorescent reporter gene (R26-TdTomato) tracking, thereby proposing GPR30 - a G protein-coupled estrogen receptor - as a novel chief cell marker.The researchers induced gastric metaplasia through high-dose tamoxifen, DMP-777, and *H. pylori* infection, while establishing Kras(G12D) and HRAS(G12V) mutant mouse models. Using dichloroacetate (DCA) to inhibit PDK activity, they investigated cellular competition in gastric mucosal renewal and homeostasis. Key findings revealed: GPR30+ chief cells were depleted upon Kras activation without triggering metaplasia/dysplasia, suggesting Kras mutation selectively impairs chief cells while neck-derived cell lineages expand via compensatory mechanisms.Mechanistically, metaplastic stimuli eliminate chief cells through GPR30/PDK-dependent cellular competition. Genetic ablation of GPR30 or pharmacological PDK inhibition preserved chief cell populations and attenuated neck lineage expansion. Metabolic reprogramming via PDK activation under metaplastic stress creates metabolic vulnerability in chief cells, ultimately excluding them as SPEM precursors. Instead, compensatory proliferation of neck progenitors emerges as the probable cellular origin of SPEM (**Figure 2**).

This study circumvented the limitations of the Cre-loxP system, enabling faster induction of specific labeling while reducing the toxic interference of tamoxifen, thereby revealing the critical role of PDK-dependent cell clearance. However, the key conclusions critically hinge on the assertion that GPR30 exclusively labels all chief cells, whereas current understanding of GPR30’s structural expression patterns remains inconsistent (100). This suggests that whether chief cells are completely labeled remains uncertain. Furthermore, some limitations of the study remain unresolved, including the lack of validation in chronic models and the undefined clinical relevance to humans. The dual-pathway regeneration strategy in gastric epithelial repair can be understood as follows: during homeostasis, isthmus stem cells replenish multiple epithelial lineages (pit, neck, and parietal cells), representing a homeostatic repair process that is slow yet precise. In contrast, chief cells in the basal region primarily maintain their homeostasis through self-replication (7). Upon acute gastric mucosal injury, the chief cell transdifferentiation pathway is activated, enabling rapid but less stable repair that predisposes to carcinogenesis (70). This paradigm has led some researchers to postulate dual stem cell niches in gastric glands - isthmic and basal compartments - potentially reconciling the enduring controversy regarding SPEM origins (chief cell vs stem cell hypotheses) and addressing ongoing debates about stem cell marker specificity (95) (**Table 1**).

**Table 1:** Summary of studies on different origins of SPEM.

Controversy	Animal model	Markers			Chief cell fate	Cite
		Parietal cell	Chief cell	SPEM cell		
Chief Cells (passive)	GIF-trAT	H/K ATPase	GIF	TFF2 GSII	SPEM	(72)
	Slc26a9^fl/fl^/Atp4b-Cre	H/K ATPase	Mist1	TFF2 MUC6 CHIA	SPEM IM Gastric cancel	(75)
	Mist1^CreER / +^	H/K ATPase	Mist1,GIF	TFF2	SPEM Mucous neck cell	(9)
	Human stomach tissue	ATP4a ATP4b	Pga3 Pga4	MUC6 TFF2 CD44 SOX	Mucous neck cell SPEM	(73)
Chief Cells (active)	Runx3^-/-^	H/K ATPase	GIF	MUC6 TFF2	SPEM	(83)
	CHIA^rox/rox^-GIF-Dre	ATP4b	Lgr5 Mist1	Wfdc2 TFF2 CD44	SPEM Mucous neck cell	(82)
Stem Cells	Lgr5-2A-CreERT, Lgr5-EGFP-IRES-CreERT	–	Lgr5 GIF Mist1	GS II	Isthmus stem cells and progenitor cells	(8)
	TetO-Cre,Gpr30-rtTA mice	H/K ATPase	GPR30 GIF	GS II	Isthmus stem cells and progenitor cells	(99)

## 4 Conclusion

Since the conceptualization of SPEM, multiple research groups have utilized chemical induction, inflammatory stimuli, and gene-editing technologies to investigate its cellular origins. This pre-metaplastic pathological stage is now gaining renewed scientific attention. Elucidating the pathogenesis of this metaplastic lesion may enable subtype-based precision intervention strategies for SPEM. While earlier studies attributed this gastric precancerous metaplasia to parietal cell loss-induced chief cell transdifferentiation, emerging perspectives emphasizing autonomous chief cell depletion and the cancer-initiating potential of stem cells challenge this paradigm. The core controversy centers on potential heterogeneity in SPEM cellular origins. The intensifying debate underscores an urgent need for dual-reporter lineage tracing models to simultaneously resolve spatiotemporal dynamics of stem/chief cell fate trajectories, complemented by clinical validation of evolutionary conservation in transformation mechanisms.

Before that, systematically consolidating current evidence to establish an interim SPEM classification framework may provide a guiding scaffold for deciphering its biological progression. By integrating existing knowledge on cellular origins, pathogenic drivers, and acute/chronic progression trajectories, this taxonomy would not only prioritize targeted tool development and optimize experimental model selection, but also propel the design of precision clinical strategies—including subtype-specific biomarker screening, personalized intervention protocols, and dynamic prognostic assessment—ultimately charting a clear roadmap for future targeted ablation technologies. Some researchers proposed classifying SPEM into three subtypes according to the acute and chronic study models and genetic drivers: acute basal SPEM (aSPEM) in chemical models, chronic SPEM (cSPEM) in inflammatory contexts, and Ras signaling-driven SPEM (rSPEM) (99). This view is a detailed categorization of the SPEM dual pathway progression, though broader consensus remains pending.
